# Stereotactic Brain Biopsy with Awake Craniotomy: Our Awake Craniotomy Experience on a Complicated Case and Mini Review

**DOI:** 10.4274/TJAR.2025.241823

**Published:** 2025-07-24

**Authors:** Can Ozan Yazar, Atakan Sezgi, Musa Zengin, Caner Ünlüer, Seyyid Furkan Kına, Emine Arık, Elif Şule Özdemir Sezgi, Jülide Ergil

**Affiliations:** 1 University of Health Sciences Türkiye, Ankara Etlik City Hospital, Clinic of Anaesthesiology and Reanimation, Ankara, Türkiye; 2 University of Health Sciences Türkiye, Ankara Etlik City Hospital, Clinic of Neurosurgery, Ankara, Türkiye

**Keywords:** Anaesthesia management, awake craniotomy, brain biopsy, pain, regional anaesthesia

## Abstract

Awake craniotomy (AC) is a surgical technique where the patient stays conscious and interacts with the surgical team throughout part or all of the brain operation. In this case report, a 71-year-old ASA-3 patient with multiple comorbidities scheduled for a stereotactic brain biopsy was treated using AC. Our experience with AC, combined with a scalp block, is described in this case. AC is a safe technique that can be applied in patients with partially impaired communication abilities and may be particularly beneficial for those with multiple chronic conditions.

**Main Points**

• Awake craniotomy (AC) describes a surgical approach where the patient remains conscious for a portion or the entirety of the procedure, actively engaging with the surgical team.

• Thanks to the AC performed with the scalp block, the neurological examination of the patient can be performed during surgery. Thus, the success rate of the operation increases.

• Scalp block anaesthesia is a safe approach that can be preferably applied, especially in cases where general anaesthesia poses risks of cardiorespiratory depression during induction, maintenance, and emergence.

## Introduction

Awake craniotomy (AC), which was first performed in 1886 by Sir Victor Horsley to localize epileptic foci, describes a surgical approach where the patient remains conscious for a portion or the entirety of the procedure, actively engaging with the surgical team.^1^ Indications for AC include tumor removal near the motor and sensory cortex, deep brain stimulation (DBS) surgery, and surgical interventions such as ventriculostomy and thalamotomy. Relative contraindications include anxiety disorders, marked dysphagia, confusion or somnolence, substance or alcohol addiction, restless leg syndrome, chronic pain conditions, low pain tolerance, obstructive sleep apnea, morbid obesity, and uncontrolled coughing.

Conscious sedation is desired in AC. During AC, the patient must respond appropriately to verbal/tactile stimuli, maintain an unobstructed airway without intervention, and exhibit adequate ventilation.^2^

AC is often preferred over general anaesthesia due to the latter’s undesirable side effects. In AC, the primary goals are to optimize interventions in target areas and prevent neurological deficits to enhance survival and maintain quality of life. Communication is crucial for the success of AC, and the inability to communicate can be considered a relative contraindication. Contrary to common knowledge, we successfully performed AC in this case on a patient with limited communication abilities and a Glasgow Glasgow Coma Scale (GCS) score of 12. Our primary objective was not cortical protection but rather shielding a high-risk patient with multiple comorbidities from the hemodynamic side effects of general anaesthesia. Through this case report, in which we share our AC experience, we aim to demonstrate that AC can be a viable alternative to general anaesthesia in similar patients.

## Case Report

**Figure 1:**
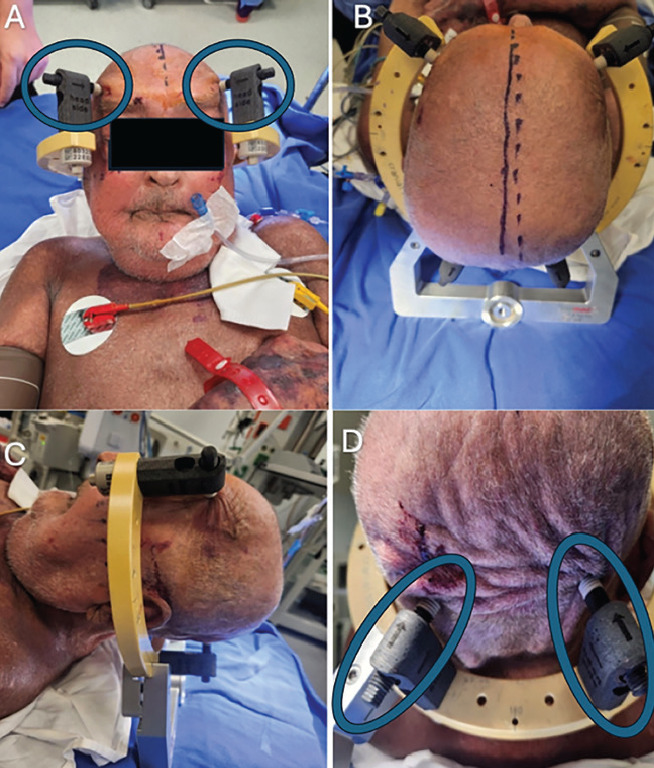
Mayfield Pins head clamp placement. A: Supine, B: Craniocaudal, C: Lateral, D: Posterior views. The placement of the pins is indicated by blue circles. The head was stabilized using a total of four pins.

**Figure 2:**
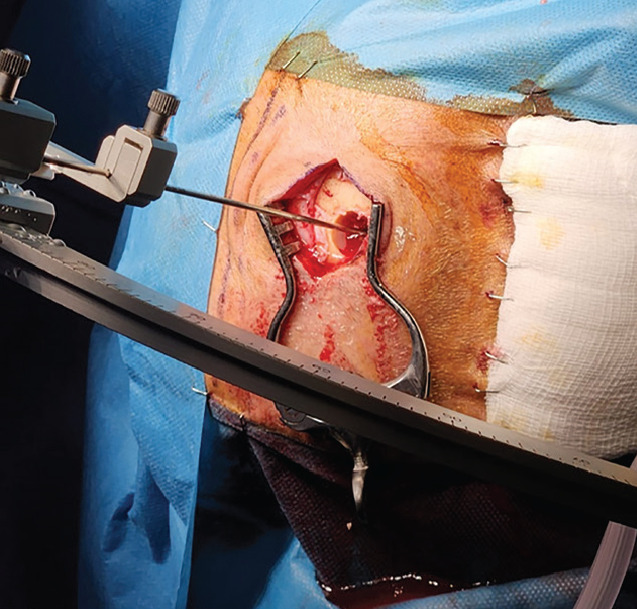
Stereotactic brain biopsy application.

A 71-year-old male patient was scheduled for a stereotactic brain biopsy operation due to a mass in the brain. His anamnesis included hypertension, chronic renal failure, and Stevens-Johnson Syndrome. She had undergone a mass excision operation for brain 42 years ago. On physical examination, the GCS was evaluated as 12. The patient opened their eyes in response to verbal stimuli and localized pain. However, they had limited orientation and were confused. Preoperative hemodialysis was planned because of a creatinine of 2.42 mg dL^-1^, a glomerular filtration rate below 30 mL min^-1^ 1.73 m^2^ and potassium of 6.5 mmol L^-1^. The patient was accepted as American Society of Anaesthesiologists (ASA) physical status scale 3 risk, and a postoperative intensive care unit was prepared. A preoperative 8-hour solid and liquid diet was administered. The patient was taken to the operating room table, and routine monitoring was performed (electrocardiography, non-invasive blood pressure measurement, peripheral pulse saturation measurement). Patient compliance was not adequate, and cooperation and orientation were limited. Dexmedetomidine at a dose of 1 µg kg^-1^ was given by intravenous infusion at 15 minutes for sedation before the scalp block. During dexmedetomidine administration, consciousness level, GCS, and hemodynamic parameters were closely monitored. No additional doses of dexmedetomidine were administered. For scalp block application, 40 mL of the drug was prepared as 36 mL of 0.5% bupivacaine and 4 mL of 1:200,000 epinephrine. Then, this local anaesthetic (LA) solution was divided into supratrochlear (2.5 mL), supraorbital (2.5 mL), zygomaticotemporal (2.5 mL), auriculotemporal (2.5 mL), greater (5 mL), and lesser occipital nerve (3 mL) regions bilaterally for scalp anaesthesia. After adequate anaesthesia was achieved, the Mayfield Pins head clamp was placed, and the brain was taken to computed tomography (CT) for brain mapping (Figure 1). After the CT scan, the patient returned to the operating theater and was placed on the table and given a supine position. A nasal cannula was fixed to the patient’s cheek, and oxygen support was provided. Intravenous nicardipine infusion was initiated when the noninvasive blood pressure measured 30 minutes after the start of surgery was 167/98 mmHg. Blood pressure was controlled with a nicardipine infusion (below 140/90 mmHg). The surgical team drilled a hole in the cranial bone for stereotactic brain biopsy and sent the biopsy needle to the relevant cortical region (Figure 2). The patient was awake and had limited communication throughout the operation. No deterioration in neurological status was observed. Vital signs were stable. The operation was completed without complications. The patient, with stable vital signs and a GCS of 12, was transferred to the intensive care unit postoperatively.

## Discussion

AC is commonly employed for procedures such as cranial mass excision, DBS, ventriculostomy, and thalamotomy. A stereotactic biopsy procedure with AC was performed on a patient with concomitant chronic diseases and limited communication skills. We tried to explain the scalp block application, the stages of AC, the points to be considered, complications, and management through this case.

Relative contraindications to AC include anxiety disorders, marked dysphagia, confusion, and somnolence. Patients with high comorbidity and poor communication skills are not preferred in the routine selection of AC cases. On the other hand, in some cases, AC may be preferred to avoid possible complications of GA for high-risk patients. In a case report by Heifets et al.,^3^ it was demonstrated that a successful AC was performed in a high-risk patient diagnosed with Eisenmenger syndrome, avoiding the risks associated with GA. D’Antico et al.^4^ also similarly preferred AC in a patient with cyanotic congenital heart disease who underwent emergency craniotomy due to cerebral abscess. The patient had a history of bacterial endocarditis, pulmonary hemorrhage, renal and splenic infarctions, transient ischemic attack, and recurrent supraventricular tachycardia. The cerebral abscess drainage was completed without complications under AC. Meng et al.^5^ described successful anaesthetic management of AC in a patient with cardiomyopathy and low cardiac output. Sethi and Kapil.^6^ preferred to use AC in brain abscess surgery to maintain hemodynamic stability and prevent increased right-to-left shunting in a child with uncorrected tetralogy of Fallot. The procedure was performed without complications. The reason for choosing AC in our case was the possibility of not tolerating GA induction and even the risks of the waking period rather than conscious sedation. In our patient in the ASA 3 risk group, we completed AC surgery with a scalp block without complications.

Mapping of high-level cognitive functions is critical during AC surgery. Therefore, the depth of sedation should be at a level that does not disrupt cortical mapping. Therefore, sedative agents used in AC operations should be carefully selected. The standard protocol for sedation in AC remains the combination of propofol and remifentanil. Several studies have compared the combination of propofol and remifentanil or propofol alone with dexmedetomidine. A recent study comparing propofol-remifentanil with dexmedetomidine found that dexmedetomidine was associated with fewer intraoperative respiratory complications.^7^ The primary disadvantage of the propofol-remifentanil combination is the potential for delirium during the awakening phase. A meta-analysis comparing dexmedetomidine with propofol alone demonstrated a strong association between dexmedetomidine use and surgical satisfaction.^8^ Propofol has limitations, such as prolonged emergence time and interference with intraoperative brain mapping. Another study found that the dexmedetomidine group had a shorter wake-up time compared to the propofol group.^9^ Although high doses of dexmedetomidine may cause bradycardia, such high doses are not required in AC. Additionally, dexmedetomidine may be beneficial for long-duration surgeries due to its suitability for prolonged sedation.^10^ In epilepsy surgeries, the combination of propofol and dexmedetomidine has been compared with the combination of propofol and remifentanil during AC. No significant difference was found between the combination of propofol and dexmedetomidine and the combination of propofol and remifentanil groups in terms of patient satisfaction. However, the incidence of nausea, vomiting, and respiratory depression was statistically higher in the combination of propofol and remifentanil group.^11^ The reasons for choosing dexmedetomidine for sedation in our case are that it is rapidly metabolized, has no residual effect, and does not impair the hemodynamic response in the intraoperative period.

**Figure 3:**
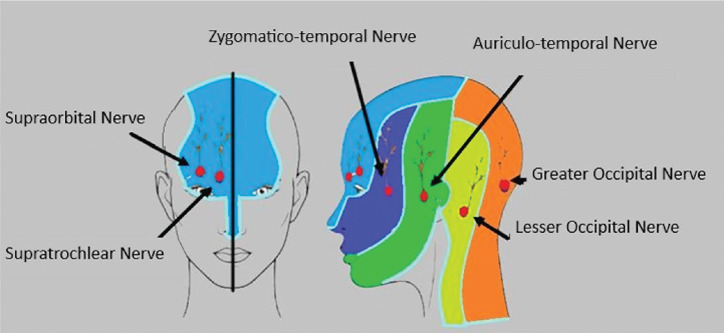
Sensory innervation of the skin of the head and neck.

The first stimulating parts of the procedure, such as the application of Mayfield Pins, skin incision, and bone flap removal, are painful. After sedation and before securing the head with Mayfield pins, a scalp block is typically administered bilaterally to ensure effective analgesia during AC. The primary advantage of the scalp block is that most of the nerves supplying the scalp are superficial terminal sensory branches, resulting in a lower risk of nerve damage compared to deeper motor nerves. Six nerves (supratrochlear, supraorbital, auriculotemporal, temporozygomatic, greater occipital and lesser occipital nerves) (Figure 3) are blocked bilaterally for a complete scalp block. Approximately 40 mL of LA is required. To extend the duration of action and minimize systemic absorption, the LA should be combined with 1:200,000 epinephrine. If additional dosing is necessary, one-quarter of the initial dose can be administered 2-4 hours after the first dose, half of the initial dose 4-8 hours later, and a full dose can be repeated 8 hours after the initial administration.^12^ A scalp block can also include a great auricular nerve block. The great auricular nerve, the largest ascending branch of the cervical plexus, arises from the C2 and C3 spinal nerves. Its posterior branch provides sensory innervation to the mastoid process and the skin behind the auricle.

Anaesthesia during AC is primarily achieved through scalp nerve blocks with LA agents. Bupivacaine is one of these agents. Bupivacaine with epinephrine at a 0.5% concentration has an onset of action of 15-30 minutes, while the anaesthetic duration ranges from 5 to 15 hours. The metabolism of LAs varies depending on whether they are ester- or amide-based. Ester LAs are metabolized by plasma cholinesterase, whereas amide LAs are metabolized by hepatic enzymes. In the presence of hepatic diseases, the dosage of amide LAs, such as bupivacaine, should be reduced for repeated or continuous administration. Bupivacaine is not metabolized by the kidneys; however, in conditions that reduce hepatic blood flow, such as renal or cardiac disease, the clearance of LAs may decrease, leading to a prolonged duration of action.^13^ LA use should be carefully considered in similar patients. In our patient, due to the presence of renal failure, the administered dose was kept below the maximum allowable dose.

Positioning the AC patient is an important step before handing it over to the surgical team. The most commonly preferred positions are supine, semi-sitting, and lateral positions. It is important to give the sniffing position during the fixation of the head to the head clamps to facilitate access to the airway. When positioning the head, care should be taken not to over-curl the neck to reduce pressure on the neck vessels.^12^ In addition to facilitating access to the surgical field, the semi-lateral position increases face-to-face interaction in the awake patient while facing the anaesthesia workstation and alleviates the respiratory workload, especially in obese patients. Especially in epilepsy surgery, patient communication facilitates the observation of the anaesthetist in terms of seizure monitoring. In our case, the patient was given a supine position due to the location of the surgical procedure, the neck was relieved, and easy airway access was provided.

Undesirable conditions such as snoring and upper airway obstruction may occur in patients undergoing AC. For this reason, continuous positive pressure can be applied with the anaesthesia circuit to reduce vibration in the surgical field. In a randomized controlled study of 65 patients undergoing AC, patients were given humidified high-flow nasal cannula (HFNC) airway management, and the safety and efficacy of this method were evaluated. Patients using HFNC with an oxygen flow rate of 40 or 60 L min showed less airway obstruction and injuries.^14^ In our case, we provided oxygen support with a nasal cannula and did not observe any complications.

In cranial surgeries, urinary catheterization is generally necessary in terms of urine output and fluid balance monitoring. However, in some cases, surgery can be performed without urinary catheterization. In a study by Ozlu et al.,^15^ a total of 26 patients who underwent DBS and an AC procedure did not receive a urinary catheter. Foley catheter applications are not frequently preferred because the operation time is short and the patient is awake. However, it may be considered in operations lasting longer than 4 hours and intraoperative mannitol administration.^12^ Intraurethral lidocaine administration may be preferred for catheter insertion and the discomfort that may occur afterward. We did not use a urinary catheter in our case because the estimated surgical time was less than 4 hours.

During AC, potential complications include seizures, hypertension, nausea-vomiting, air embolism, and hyponatremia. Nausea has been reported in approximately 4% to 18.4% of cases and may be precipitated by surgical stimulation, particularly during manipulation of the dura or cerebral vessels, as well as by the use of opioids or the presence of anxiety.^16^ Vomiting is rare and managed with antiemetics like ondansetron (4 mg); dexmedetomidine and propofol offer additional antiemetic benefits. Hypertension is treated with vasodilators or beta-blockers, with esmolol shown to provide intraoperative hemodynamic stability.^16^ Airway obstruction due to hypoventilation or hypercarbia is managed by reducing sedation, encouraging deep breaths, or providing mask ventilation; uncooperative patients may require general anaesthesia. Elevating the head 30° helps prevent venous congestion, while hyperosmotic therapy and normocarbic ventilation support respiratory function. Seizures, occurring in 2-20% of cases during brain mapping, are usually brief and self-limiting. Initial treatment involves brain irrigation with cold saline; if needed, propofol (10-20 mg IV) or midazolam (1-2 mg IV) can be used, with propofol preferred for electrocorticography.^12^  Levetiracetam (500 mg IV) may be given prophylactically in patients not on antiepileptic drugs. The most critical risk in the sitting position is venous air embolism due to negative intravascular pressure.^12^ This can lead to pulmonary vasoconstriction, decreased EtCO₂, increased PaCO₂, hypoxemia, arrhythmias, and chest pain. As embolism severity increases, hypotension and tachycardia may occur due to increased right heart strain.^17  ^Treatment includes Trendelenburg positioning, aspiration via central venous catheter, and vasopressors such as ephedrine, dobutamine, or norepinephrine.

In AC, procedures associated with general anaesthesia, such as endotracheal intubation and mechanical ventilation, can be avoided, leading to reduced hemodynamic and physiological disturbances. Additionally, compared to craniotomy under GA, postoperative pain, nausea, and vomiting are less frequently observed.Studies have compared AC with GA for craniotomy. Both applications have their advantages and disadvantages (Table 1).^18^ One study supported the superiority of AC in terms of neurological outcomes and resection quality in supratentorial mass excisions.^19^ A recent meta-analysis examining AC and GA in glioblastoma surgeries found significant evidence favoring AC, with a notably longer median postoperative survival.^20^ Consequently, hypotension and the need for vasopressors are less common compared to general anaesthesia. Moreover, AC is associated with a shorter hospital stay, potentially reducing the risks of hospital-acquired infections and deep vein thrombosis.

### Study Limitations

This case report has some limitations. Firstly, our report consists of a single case, and generalizations to the whole population cannot be made. Therefore, randomized controlled trials are needed in this regard. Secondly, after the scalp block, only the surgical area was checked, and no more detailed dermatomal examination was performed. Thirdly, our patient could not be monitored with a BIS monitor. Although we prefer to measure the depth of sedation with the Ramsey Sedation Scale, BIS monitoring gives more quantitative values.

## Conclusion

In conclusion, it has been demonstrated that AC can be a viable method not only for functional cortical mapping but also for fragile patients with high comorbidities, limited communication, and low GCS. Scalp block anaesthesia is a safe approach, particularly in cases where GA poses a risk of cardiorespiratory depression during induction, maintenance, and recovery.

## Ethics

**Informed Consent:** Written consent has been obtained from the patient indicating his approval for publication.

## Figures and Tables

**Table 1. Comparison of General Anaesthesia and Awake Craniotomy:** 

**Parameters**	**Awake craniotomy (AC)**	**General anaesthesia (GA)**
Airway management	Nasal cannula	Endotracheal intubation/Laringeal mask airway
Haemodynamic effects	Comparatively more stable	There is a risk of cardiorespiratory depression
Postoperative complications (nausea, pain, hypertension, tachycardia)	Less compared to GA	More often
Advantages	- Better protection of intraoperative motor and speech functions. - Reduced hospital stay and therefore reduced risk of hospital-acquired infections. - Reduction of neurological deficits after surgery. - Less need for vasopressors.	- Intraoperative safe airway management due to endotracheal intubation/LMA. - To be able to perform the necessary interventional procedures for bleeding management, fluid volume balance monitoring, arterial blood pressure monitoring.
Disadvantages	- Difficult intraoperative airway management. - Possibility of side effects due to local anaesthetics (LAST).	- Inability to perform an intraoperative examination. - Greater risk of neurological deficits. - Adverse effects on immunity associated with general anaesthesia. - The risk of hypotension and the need for vasopressors are greater after induction.

AC, awake craniotomy; GA, general anaesthesia; LAST, local anaesthesia systemic toxicity; LMA, laryngeal mask airway.
